# *S*-Petasin, the Main Sesquiterpene of *Petasites formosanus,* Inhibits Phosphodiesterase Activity and Suppresses Ovalbumin-Induced Airway Hyperresponsiveness

**DOI:** 10.1093/ecam/nep088

**Published:** 2011-02-10

**Authors:** Chung-Hung Shih, Tzu-Jung Huang, Chien-Ming Chen, Yun-Lian Lin, Wun-Chang Ko

**Affiliations:** ^1^Department of Internal Medicine, Taipei Medical University Hospital, Taiwan; ^2^Graduate Institute of Pharmacology, College of Medicine, Taipei Medical University, Taipei 110, Taiwan; ^3^Department of Medical Technology, College of Medicine, Taipei Medical University, Taiwan; ^4^National Research Institute of Chinese Medicine, Taipei, Taiwan

## Abstract

*S*-Petasin is the main sesquiterpene of *Petasites formosanus*, a traditional folk medicine used to treat hypertension, tumors and asthma in Taiwan. The aim of the present study was to investigate its inhibitory effects on phosphodiesterase (PDE) 1–5, and on ovalbumin (OVA)-induced airway hyperresponsiveness (AHR) in a murine model of allergic asthma. *S*-Petasin concentration-dependently inhibited PDE3 and PDE4 activities with 50% inhibitory concentrations (IC_50_) of 25.5, and 17.5 *μ*M, respectively. According to the Lineweaver-Burk analysis, *S*-petasin competitively inhibited PDE3 and PDE4 activities with respective dissociation constants for inhibitor binding (*K*_i_) of 25.3 and 18.1 *μ*M, respectively. Both IC_50_ and *K*_i_ values for PDE3 were significantly greater than those for PDE4. *S*-Petasin (10–30 *μ*mol/kg, administered subcutaneously (s.c.)) dose-dependently and significantly attenuated the enhanced pause (*P*_enh_) value induced by methacholine (MCh) in sensitized and challenged mice. It also significantly suppressed the increases in total inflammatory cells, lymphocytes, neutrophils, eosinophils and levels of cytokines, including interleukin (IL)-2, IL-4 and IL-5, tumor necrosis factor (TNF)-*α* and interferon (IFN)-*γ* in bronchoalveolar lavage fluid (BALF) of these mice. In addition, *S*-petasin (10–30 *μ*mol/kg, s.c.) dose-dependently and significantly attenuated total and OVA-specific immunoglobulin E (IgE) levels in the serum and BALF, and enhanced the IgG_2a_ level in serum of these mice. The PDE4_H_ value of *S*-petasin was >300 *μ*M; therefore, its PDE4_H_/PDE4_L_ value was calculated to be >17. In conclusion, the present results for *S*-petasin at least partially explain why *Petasites formosanus* is used as a folk medicine to treat asthma in Taiwan.

## 1. Introduction

Recently, Ze 339, an extract of *Petasites hybridus* L. (Compositae), was approved by the Swiss government agency, Swissmedic, as an anti-allergic drug (Tesalin; Zeller AG, Romanshorn, Switzerland) to treat seasonal allergic rhinitis. In a study by Schapowal [1], the clinical effects of Ze 339 were similar to those of cetirizine, an antagonist of histamine receptor subtype 1, although Ze 339 was reported to elicit no skin test reactivity induced by different stimuli [2]. The plant is used as a therapeutic spasmolytic agent for gastrointestinal tract spasms [3] and asthmatic attacks [4] in Europe. Four sesquiterpenoid substances, petasin, isopetasin, *S*-petasin and *S*-isopetasin, were isolated from the plant [5], and petasin was reported to have the highest spasmolytic activity [6]. These four sesquiterpenes are also present in the aerial part of *Petasites formosanus* Kitamura, a traditional folk medicine used to treat hypertension, tumors and asthma in Taiwan [7], and *S*-petasin is the most abundant [8]. *S*-Petasin, with an IC_50_ of <10 *μ*M, was proven to be the most potent in relaxing guinea pig trachea precontracted by histamine, carbachol (CCh), KCl or leukotriene (LT) D_4_, although *S*-isopetasin (IC_50_≅ 10 *μ*M) has a similar relaxing potency on CCh and KCl, but almost no effect on histamine and LT D_4_ [9]. Recently, we reported that *S*-isopetasin has bronchodilatory effects on obstructive airway hyperresponsiveness (AHR) via its antimuscarinic M_3_ antagonism [10]. *S*-Petasin, but not *S*-isopetasin, inhibited cAMP-phosphodiesterase (PDE) activity [11].

PDEs are classified according to their primary protein and complementary (c)DNA sequences, co-factors and substrate specificities, and pharmacological roles. It is now known that PDEs comprise of at least 11 distinct enzyme families that hydrolyze cAMP and/or cGMP [12]. PDE1–5 isozymes, which are calcium/calmodulin-dependent (PDE1), cGMP-stimulated (PDE2), cGMP-inhibited (PDE3), cAMP-specific (PDE4) and cGMP-specific (PDE5), have been found to be present in the canine trachea [13], guinea pig lung [14] and human bronchi [15]. PDE3 and PDE4 were identified in the guinea pig airway [16], but other isozymes might also be present. Rolipram, a prototype PDE4 selective inhibitor, has high (PDE4_H_) and low (PDE4_L_) affinities for PDE4, respectively. In general, it is believed that inhibition of PDE4_H_ is associated with an adverse response, such as nausea, vomiting and gastric hypersecretion, and inhibition of PDE4_L_ is associated with anti-inflammatory and bronchodilating effects. Therefore, the therapeutic ratio of selective PDE4 inhibitors for use in treating asthma and chronic obstructive pulmonary disease (COPD) is defined as the PDE4_H_/PDE4_L_ ratio [17, 18]

Rolipram or zardaverin (dual PDE3/4 inhibitor), but not siguazodan (a selective PDE3 inhibitor), markedly inhibited aerosol ovalbumin (OVA)-induced broncho-constriction in conscious guinea pigs, and inhibited OVA-induced contractions of isolated guinea pig trachea [19]. Underwood et al. [19] suggested that the combined inhibition of both PDE3 and PDE4 isozymes acts in an additive or synergistic manner to inhibit brochospasms in the guinea pig, although selective PDE3 or PDE4 inhibitors are ineffective against the exogenous histamine- and LTD_4_-induced contractions. The aim of present study was to investigate whether *S*-petasin inhibits both PDE3 and PDE4 isozymes, and whether it has the potential for use in treating asthma or COPD.

## 2. Methods

### 2.1. Reagents and Animals

**Figure 1:**
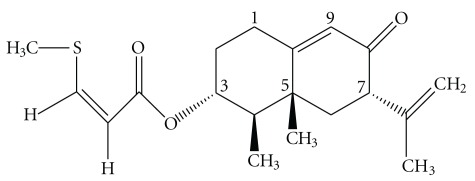
Chemical structure of *S*-petasin (mol. wt., 334), isolated from *Petasites formosanus* Kitamura.

*S*-Petasin (Figure 1) was isolated as previously described [8] from the aerial parts of *Petasites formosanus* Kitamura, and identified by spectral methods, including infrared (IR), mass spectroscopy (MS), one-dimensional (1D)- and 2D-nuclear magnetic resonance (NMR) spectroscopic techniques. The purity of *S*-petasin was >99%. Its optical rotation value was [*α*]^25^_D_ + 58.0° (c 1.0, MeOH). OVA, methacholine (MCh), polyethyleneimine, (2-hydroxypropyl)-*β*-cyclodextrin (HP*β*CD), calmodulin, dimethylsulfoxide (DMSO), Trizma HCl, bis(2-hydroxyethyl)aminotris(hydroxymethyl)methane (Bis-Tris), benzamidine, phenylmethanesulfonyl fluoride (PMSF), polyethyleneimine, d,l-dithiothreitol, ethylenediaminetetraacetic acid (EDTA), bovine serum albumin (BSA), adenosine 3′,5′ cyclic monophosphate (cAMP), guanosine 3′,5′ cyclic monophosphate (cGMP), calmodulin, Dowex resin, *Crotalus atrox* snake venom, xylazine and ketamine were purchased from Sigma Chemical (St. Louis, MO, USA). Vinpocetine, *erythro*-9-(2-hydroxy-3-nonyl)-adenine HCl (EHNA), milrinone, rolipram, 4-(3-butoxy-4-methoxybenzyl)-2-imidazolidinone (Ro 20-1724), and zaprinast were purchased from Biomol (Plymouth Meeting, PA, USA). Freund's adjuvant (*Mycobacterium butyricum*) was purchased from Pierce Biotechnology (Rockford, IL, USA). Mouse T helper (Th)1/Th2 cytokine CBA kits, and mouse IgE and IgG_2a_ ELISA sets were purchased from Pharmingen (San Diego, CA, USA). Polyethyleneglycol (PEG) 400 and ethyl alcohol were purchased from Merck (Darmstadt, Germany). [2,8-^3^H]-cAMP, [8-^3^H]-cGMP, and [*methyl*-^3^H]-rolipram were purchased from Amersham Pharmacia Biotech (Buckinghamshire, UK). Other reagents, such as CaCl_2_, MgCl_2_ and NaCl, were of analytical grade. *S*-Petasin and vinpocetin were dissolved in a mixture of ethyl alcohol and DMSO (1 : 1). EHNA and Ro 20-1724 were dissolved in ethyl alcohol. Milrinone and zaprinast were dissolved in DMSO. Other drugs were dissolved in distilled water. The final concentration of ethyl alcohol or DMSO was ≤0.5%, and did not significantly affect the activities of PDE isozymes or tracheal contractions.

Male Hartley guinea pigs (500–600 g) and female BABL/c mice at 8–12 weeks were obtained from the Animal Center of the National Science Council (Taipei, Taiwan). The animals were housed in ordinary cages at 22 ± 1°C with a humidity of 50–60% under a constant 12/12-h light/dark cycle and provided with food and water *ad libitum*. Under a protocol approved by the Animal Care and Use Committee of Taipei Medical University, the following *in vivo* and *in vitro* experiments were performed.

### 2.2. Inhibition on PDE Activities and the Lineweaver-Burk Analysis

Activities of PDE1–5, partially separated from guinea pig lungs and hearts according to the method described by Ko et al. [20], were measured with a two-step procedure according to the method of Thompson and Appleman [21], using cAMP with [^3^H]-cAMP or cGMP with [^3^H]-cGMP as substrates. The enzyme p*μ*reparation (25 *μ*L) was incubated for 30 min at 37°C in a total assay volume of 100 L containing 50 mM Tris-HCl (pH 7.4), 3 mM MgCl_2_, 1 mM dithiothreitol, 0.05% BSA, 1 *μ*M cAMP with 0.2 *μ*Ci [^3^H]-cAMP as a substrate alone or in the presence of 0.1 units calmodulin with 10 *μ*M CaCl_2_ or 5 *μ*M cGMP, and 1 *μ*M cGMP with 0.2 *μ*Ci [^3^H]-cGMP as another substrate alone or in the presence of 0.1 units calmodulin with 10 *μ*M CaCl_2_. In tests of enzyme inhibition, the reaction mixture contained 10 *μ*L of vehicle or inhibitors, at various concentrations of *S*-petasin or selective PDE1–5 inhibitors, such as vinpocetin [22], EHNA [23], milrinone [24], Ro 20-1724 [25] and zaprinast [26], as reference drugs. The reagents and homogenate were mixed on ice, and the reaction was initiated by transferring the mixture to a water bath at 37°C. Following a 30-min incubation, the reaction was stopped by transferring the reaction vessel to a bath of boiling water for 3 min. After cooling on ice, 20 *μ*L of a 1 mg/mL solution of *C. atrox* snake venom was added to the reaction mixture, and the mixture was incubated at 37°C for 10 min. Unreacted [^3^H]-cAMP or [^3^H]-cGMP was removed by the addition of 500 *μ*L of a 1-in-1 Tris-HCl (40 mM) buffer suspension of Dowex resin (1 × 8-200) with incubation on ice for 30 min. Each tube was then centrifuged for 2 min at 3700 g, and 150 *μ*L of the supernatant was removed for liquid scintillation counting. Less than 10% of the tritiated cyclic nucleotide was hydrolyzed in this assay. The total protein in each fraction used was assayed according to the method described by Bradford [27]. The PDE activities are shown as nmol/mg/min in the Lineweaver-Burk analysis.

### 2.3. Determination of PDE4_H_ Values

When the above-described guinea pigs were sacrificed, the whole brains were removed and homogenized with a glass/Teflon homogenizer (Glas-Col, Terre Haute, IN, USA) in 10 volumes of cold medium (pH 6.5) containing 20 mM Bis-Tris, 2 mM benzamidine, 2 mM EDTA, 50 mM sodium chloride, 0.1 mM PMSF and 1 mM dithiothreitol. At 4°C, the homogenate was centrifuged at 170 g for 5 min to remove connective tissues and blood vessels. The suspended homogenate was then re-centrifuged at 40 000 g for 30 min to separate the cytosolic and particulate portions. The particulate portion was re-suspended in a suspension at a concentration of 400 mg/mL (wet weight/volume), after washing three times with homogenizing buffer. The particulate portion mainly consisted of cell membranes. The binding ability of *S*-petasin (3–300 *μ*M) or Ro 20-1724 (1–10,000 nM), a reference drug, to high-affinity rolipram binding sites (HARBSs) of the membranes was determined by replacing 2 nM [^3^H]-rolipram in a reaction buffer at 30°C for 1 h, according to the method described by previous investigators [28, 29] and modified by us. Briefly, the reaction buffer consisted of 50 mM Tris-HCl and 5 mM MgCl_2_ (pH 7.5). The total volume of the reaction mixture was 25 *μ*L, consisting of 10 *μ*L of particulate suspension, 10 *μ*L of [^3^H]-rolipram and 5 *μ*L of *S*-petasin or Ro 20-1724. After 1 h, the reaction was terminated by moving the reaction vessel into crushed ice. Then the reaction mixture was transferred onto Whatman GF/B glass-fiber filters, which were soaked in a 0.3% polyethyleneimine solution in a mini-funnel. The reaction mixture was filtered by centrifuging at 90 g for 10 s, and the filtrate was collected into a 1.5 mL Eppendorf tube with a top adapted to the outlet of the mini-funnel. The filters were washed with 300 *μ*L of reaction buffer three times each in the same way, and transferred into 2 mL of cocktail for radiation counting (total binding) using a *β*-scintillation counter (Beckman, Fullerton, CA, USA). Non-specific binding, which was defined in the presence of 10 *μ*M Ro 20-1724, was subtracted from the total binding to yield the specific binding. The effective concentration (EC_50_) values of *S*-petasin and Ro 20-1724, at which a half of [^3^H]-rolipram bound onto HARBSs of cell membranes was displaced, were defined as the PDE4_H_ values, and these were related to any adverse effects, such as nausea, vomiting and gastric hypersecretion [30].

### 2.4. Airway Hyperresponsiveness In Vivo

Ten female BABL/c mice in each group were sensitized by an intraperitoneal (i.p.) injection of 20 *μ*g of OVA emulsified in 2.25 mg aluminum hydroxide gel in a total volume of 100 *μ*L on days 0 and 14. The mice were challenged via the airway by 1% OVA in saline for 30 min on Days 28, 29 and 30 by ultrasonic nebulization. Six weeks after the last of three primary OVA challenges, the mice were exposed to 1% OVA for 30 min by nebulization as a secondary challenge [31]. AHR was assessed on Day 74 (48 h after 1% OVA provocation) in each group. Each group of mice was subcutaneously (s.c.) injected with vehicle (control), or 30–100 *μ*mol/kg of *S*-petasin 2 h before and 6 and 24 h after OVA provocation. For comparison, sham-treated mice were sensitized but challenged with saline instead of 1% OVA (non-challenged). The vehicle, a mixture of alcohol : DMSO : 30% HP*β*CD : saline (0.5 :  0.5 : 1 : 8  , v/v), or *S*-petasin was injected at a volume of 0.01 mL/g of body weight. AHR was measured in unrestrained animals by barometric plethysmography [32] using a whole-body plethysmograph (WBP) and analyzed using software of Life Science Suite P3 Analysis Modules (Gould, LDS Test and Measurement LLC, Valley View, OH, USA). The mice were placed into the main chamber of the WBP, and the baseline enhanced pause (*P*_enh_) value was determined. Then the mice were nebulized first with phosphate-buffered saline (PBS), and subsequently with increasing doses (6.25–50 mg/mL) of MCh for 3 min for each nebulization, followed by readings of breathing parameters for 3 min after each nebulization with determination of *P*_enh_ values. Twenty-four hours after the *P*_enh_ determination, these mice were anesthetized with pentobarbital (50 mg/kg, i.p.), and lavaged via a tracheal tube with PBS (1 × 1.0 mL, 37°C). After lavage, blood was collected from the jugular vein and allowed to sit so that it would coagulate. The collected bronchoalveolar lavage fluid (BALF) and coagulated blood were, respectively, centrifuged at 630 g for 7 min and at 3700 g for 10 min at 4°C, respectively. After centrifugation, the supernatants of BALF and serum were stored at −20°C until the determinations of cytokines, including interleukin (IL)-2, IL-4, IL-5, tumor necrosis factor (TNF)-*α* and interferon (IFN)-*γ* by flow cytometric methods [33] using mouse Th1/Th2 cytokine CBA kits, of total immunoglobulin E (IgE), and of total IgG_2a_ using enzyme-linked immunosorbent assay (ELISA) kits (Pharmingen, San Diego, CA, USA) according to the respective recommendations of the manufacturer. OVA-specific IgE was measured as described previously [34] with some modifications. Wells were coated with 100 *μ*L of OVA (20 *μ*g/mL) instead of the capture antibody. Levels are expressed in arbitrary units, where one arbitrary unit equals the optical density of the sample divided by the optical density of unchallenged mouse serum or BALF (standard). The pellet from BALF was re-suspended in ACK lysing buffer (1.658 g NH_4_Cl, 0.2 g KHCO_3_ and 1.44 mg EDTA in 200 mL of water) to lyse the residual erythrocytes in each sample. The number of inflammatory cells was counted using a hemocytometer (Hausser Scientific, Horsham, PA, USA). Cytospun slides were stained and differentiated in a blinded fashion by counting at least 100 cells under light microscopy.

### 2.5. Xylazine/Ketamine-Induced Anesthesia

According to the method described by Robichaud et al. [35] and modified by us, *S*-petasin (10–100 *μ*mol/kg, s.c.) or rolipram (0.01–1 *μ*mol/kg, s.c.), a reference drug, was injected into 8–12 week-old female BALB/c mice 1 h or 15 min, respectively, prior to an i.p. injection of xylazine (10 mg/kg)/ketamine (70 mg/kg). The vehicle (control) for *S*-petasin or for rolipram was a mixture of alcohol : DMSO : 30% HP*β*CD : saline (0.5 : 0.5 : 1 : 8 , v/v), or alcohol : DMSO : PEG 400 : saline (0.5 : 0.5 : 1 : 8, v/v), respectively. After loss of the righting reflex (i.e., when a mouse remained on its back and no longer spontaneously righted itself to a prone position), the duration of anesthesia was measured until its return as an endpoint [35].

### 2.6. OVA-Induced Tracheal Contractions In Vitro

Male Hartley guinea pigs (500–600 g) were sensitized by intramuscular injections of 0.7 mL of 5% (w/v) OVA in saline on Days 1, 4 and 43, and in adjuvant on Days 25 and 39 into each thigh, according to a method described by Underwood et al. [19] and modified by us. Three days after the last injection, sensitized guinea pigs were sacrificed by cervical dislocation, and their tracheas removed. Each trachea was cut into six segments. Each segment consisted of three cartilage rings. All segments were cut open opposite the trachealis. After the segments were randomized to minimize regional variability, they were tied at one end to holders via silk sutures, placed in 5 mL of normal Krebs solution containing indomethacin (3 *μ*M), gassed with a mixture of 95% O_2_ plus 5% CO_2_ at 37°C, and attached by the other end of each segment to force displacement transducers (Grass FT03, Quincy, MA, USA) for the isometric recording of tension changes on a polygraph (Gould RS3200, Cleveland, OH, USA). The composition of the normal Krebs solution was (mM): NaCl 118, KCl 4.7, MgSO_4_ 1.2, KH_2_PO_4_ 1.2, CaCl_2_ 2.5, NaHCO_3_ 25 and dextrose 10.1. Tissues were suspended in normal Krebs solution under an initial tension of 1.5 g and allowed to equilibrate for at least 1 h with washing at 15-min intervals. After the tissues were precontracted with KCl (60 mM) and washed with normal Krebs solution, OVA (0.1–100 *μ*g/mL) was cumulatively added, and contractions were allowed to reach a steady state at each concentration. To evaluate the suppressive effect of *S*-petasin on OVA-induced contractions, each tissue was preincubated with each concentration (30–300 *μ*M) of *S*-petasin or its vehicle for 15 min and then challenged with cumulative OVA again. Therefore, the log concentration-response curves of OVA were constructed in the absence and presence of *S*-petasin. The tension of the precontraction induced by KCl was set as 100%.

### 2.7. Statistical Analysis

Concentrations of test compounds at which 50% of maximum activity (IC_50_ or EC_50_ value) was produced were compared to each other. The IC_50_ and EC_50_ values were calculated using a non-linear regression analysis by the software SigmaPlot 10.0 (Sigma Chemical, St. Louis, MO, USA). All values are given as the mean ± SEM. Differences among values were statistically calculated by one-way analysis of variance (ANOVA), and then determined by Dunnett's test. The difference between two values, however, was determined by use of student's *t*-test. Differences with *P* < .05 were considered statistically significant.

## 3. Results

### 3.1. Selective and Competitive Inhibition of PDE4 by *S*-Petasin

**Figure 2:**
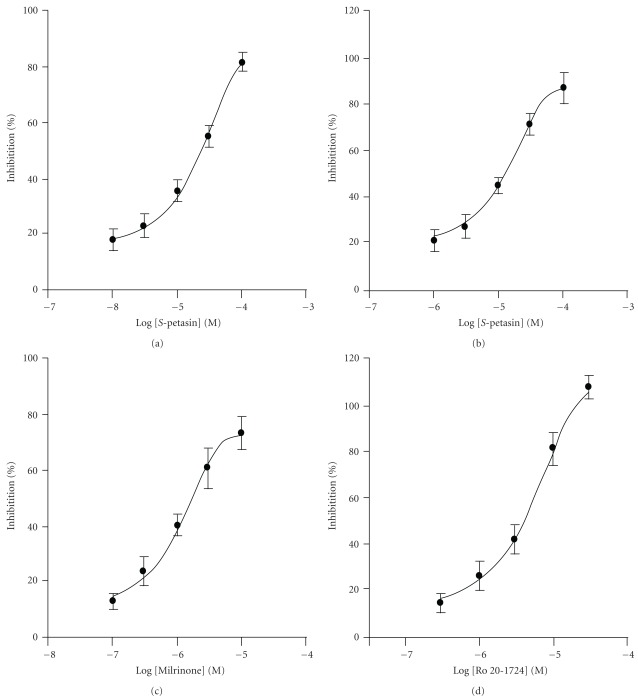
Log concentration-inhibition curves of *S*-petasin and its reference drugs on phosphodiesterase 3 (PDE3) and PDE4 activities. *S*-Petasin (a, b), milrinone, a selective PDE3 inhibitor (c), and Ro 20-1724, a selective PDE4 inhibitor (d) concentration-dependently inhibited PDE3 (a, c) and PDE4 (b, d) activities. Each value represents the mean ± SEM (*n* = 4–8).

**Figure 3:**
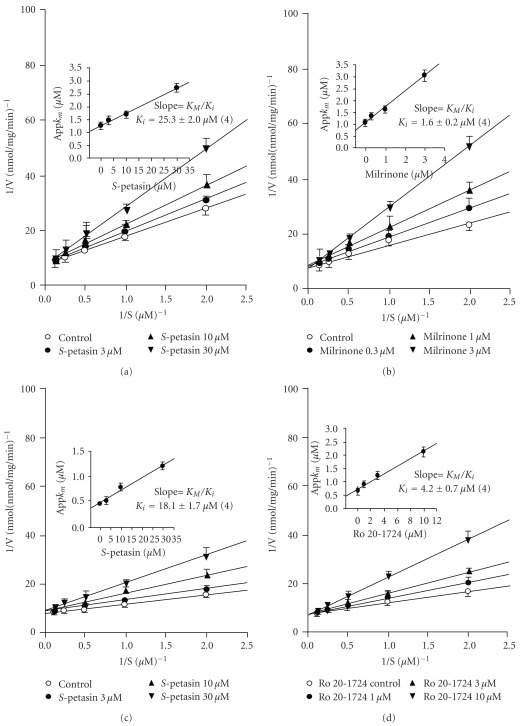
Inhibition of PDE3- (a, b) or PDE4 (c, d)-induced cAMP hydrolysis by *S*-petasin (a, c), and the reference drugs, milrinone (b) and Ro 20-1724 (d). The activities of PDE3 and PDE4 in the presence of various concentrations of *S*-petasin or reference drugs, and the substrate (cAMP) were plotted according to the Lineweaver-Burk analysis. The *K*_i_ value was determined from the equation of the apparent *K*_m_ as a function of the inhibitor concentration (inset). Each value represents the mean ± SEM (*n* = 4).

*S*-Petasin (1–100 *μ*M) concentration-dependently inhibited PDE3 (Figure 2(a)) and PDE4 (Figure 2(b)) with IC_50_ values of 25.5 ± 1.5 *μ*M (*n* = 4) and 17.5 ± 2.4 *μ*M (*n* = 6), respectively, which significantly differed from each other (Table 1).  Figure 2(c) shows the concentration-inhibition curve of milrinone, a selective PDE3 inhibitor, on PDE3, and Figure 2(d) shows that of Ro 20-1724, a selective PDE4 inhibitor, on PDE4. However, *S*-petasin did not inhibit PDE1, PDE2 or PDE5 activities (IC_50_ values > 100 *μ*M, Table 1). The IC_50_ values of all reference drugs used are shown in Table 1. According to the Lineweaver-Burk analysis, *S*-petasin (3–30 *μ*M) and milrinone (0.3–3 *μ*M) competitively inhibited PDE3 activity (Figures 3(a) and 3(b)), because the 1/*V*_max_ values were not significantly affected by various concentrations of *S*-petasin or milrinone. Their *K*_i_ values were, respectively, calculated to be 25.3 ± 2.0 (*n* = 4) and 1.6 ± 0.2 (*n* = 4) *μ*M (Figures 3(a) and 3(b), inset). Similarly, *S*-petasin (3–30 *μ*M) and Ro 20-1724 (1–10 *μ*M) competitively inhibited PDE4 activity (Figures 3(c) and 3(d)). Their *K*_i_ values were, respectively, calculated to be 18.1 ± 1.7 *μ*M (*n* = 4) and 4.2 ± 0.7 *μ*M (*n* = 4) (Figures 3(c) and 3(d), inset). The *K*_i_ value of *S*-petasin for PDE3 significantly differed from that for PDE4, suggesting that *S*-petasin has a higher affinity for PDE4 than for PDE3. The *K*_i_ values of *S*-petasin and their reference drugs are shown in Table 1. The anti-inflammatory effects of PDE4 inhibitors were reported to be associated with inhibition of PDE4 catalytic activity [36], and the anti-inflammatory effects were also correlated to PDE4_L_ inhibition [30]. Therefore, the IC_50_ values (Table 1) of *S*-petasin (17.5 *μ*M) and Ro 20-1724 (6.9 *μ*M) for inhibiting PDE4 catalytic activity were taken to be the PDE4_L_ values.

### 3.2. PDE4_H_/PDE4_L_ Ratios

**Figure 4:**
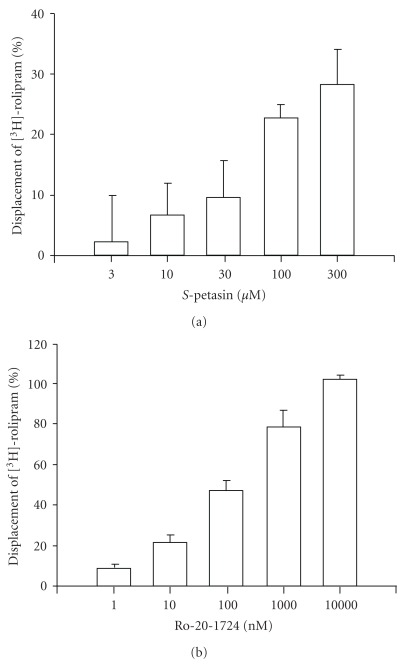
Displacement of [^3^H]-rolipram by *S*-petasin (a) and Ro 20-1724 (b) in high-affinity rolipram binding sites of guinea pig whole brain particulates. Each value represents the mean ± SEM (*n* = 6–9).

*S*-Petasin (3–300 *μ*M) concentration-dependently displaced the [^3^H]-rolipram binding on HARBSs of guinea pig brain cell membranes (Figure 4(a)). At the highest concentration (300 *μ*M), however, the percentage displacement by *S*-petasin was 28.0%±5.7% (*n* = 7). Owing to the solubility of *S*-petasin, its concentration cannot exceed 300 *μ*M. In other words, the EC_50_ value of *S*-petasin for the displacement was >300 *μ*M. Ro 20-1724 (1–10 000 nM), a selective PDE4 inhibitor, also concentration-dependently displaced [^3^H]-rolipram binding on HARBSs (Figure 4(b)). In contrast to *S*-petasin, the percentage of the displacement by Ro 20-1724 at the highest concentration (10 000 nM) was 100%±2.1% (*n* = 9). The EC_50_ value of Ro 20-1724 for displacement was 95.8 ± 13.6 nM (*n* = 9). According to the definition (see Section 2), the PDE4_H_ values of *S*-petasin and Ro 20-1724 were >300 *μ*M and 95.8 ± 13.6 nM, respectively. Thus, the PDE4_H_/PDE4_L_ values of *S*-petasin and Ro 20-1724 were calculated to be >17 and 0.014, respectively.

#### 3.3. Suppression of AHR In Vivo

**Figure 5:**
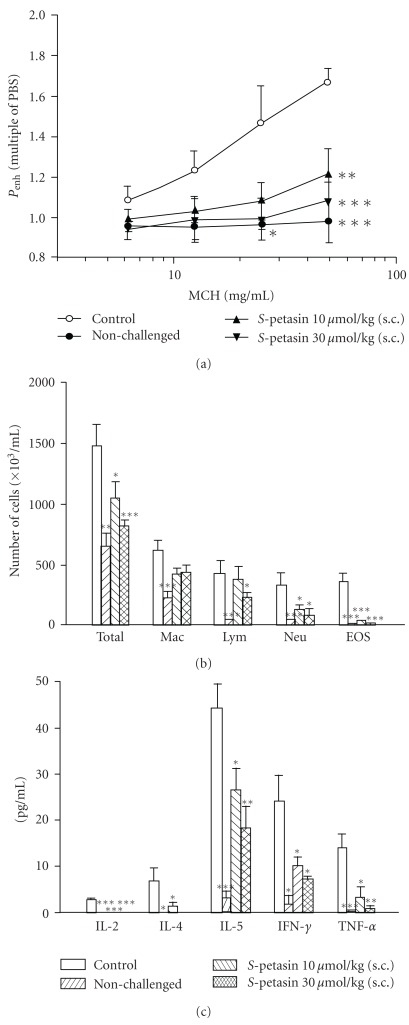
Effect of *S*-petasin (10–30 *μ*mol/kg, s.c.) on *P*_enh_ (a), inflammatory cells (b), and cytokines (c) in sensitized mice which received aerosolized MCh (6.25–50 mg/mL) 2 days after secondary allergen challenge. *P < .05, **P < .01, and ***P < .001, compared to the vehicle (control). The number of mice in each group was 10. Total, total cells; Mac, macrophages; Lym, lymphocytes; Neu, neutrophils; Eos, eosinophils; IL, interleukin; TNF-*α*, tumor necrosis factor-*α*; TNF-*γ*, tumor necrosis factor-*γ*.

*P*_ enh_ values at the baseline for control (vehicle), non-challenged and 10 and 30 *μ*mol/kg *S*-petasin subcutaneously injected groups were 3.04 ± 0.31, 2.95 ± 0.31, 3.19 ± 0.47 and 3.13 ± 0.32, respectively, and these values did not significantly differ from each other. *P*_enh_ values of PBS nebulization for each group were 2.87 ± 0.12, 2.82 ± 0.06, 2.99 ± 0.22 and 3.02 ± 0.23, respectively, which also did not significantly differ from each other. Administration of nebulized PBS did not affect the *P*_enh_ value of the baseline in each group. However, MCh (6.25–50 mg/mL) concentration-dependently increased *P*_enh_ values from 1.08 ± 0.08-fold of PBS exposure to 1.67 ± 0.07-fold in control sensitized and challenged mice (Figure 5(a)). *S*-Petasin (10–30 *μ*mol/kg, s.c.) dose-dependently and significantly attenuated the enhancement of the *P*_enh_ value induced by MCh at 50 mg/mL. The *P*_enh_ value of MCh (50 mg/mL) in non-challenged mice was almost unchanged compared to that of PBS nebulization and was significantly less than that in control sensitized and challenged mice (Figure 5(a)).

#### 3.4. Suppression of Inflammatory Cells in BALF

Total inflammatory cells, macrophages, lymphocytes, neutrophils and eosinophils from the BALF of control sensitized and challenged mice significantly increased compared to non-challenged mice (Figure 5(b)). *S*-Petasin (10–30 *μ*mol/kg, s.c.) also significantly suppressed the increases in total inflammatory cells, lymphocytes, neutrophils and eosinophils, with the exception of lymphocytes at 10 *μ*mol/kg (Figure 5(b)). Unexpectedly, macrophages were not affected by *S*-petasin treatment.

#### 3.5. Suppression of Cytokines in BALF

Compared to non-challenged mice, the levels of cytokines, such as IL-2, IL-4, IL-5, IFN-*γ* and TNF-*α* in the BALF of control sensitized and challenged mice significantly increased (Figure 5(c)). *S*-Petasin (10–30 *μ*mol/kg, s.c.) also significantly suppressed increases in the levels of these cytokines, with the exception of IL-4 at a dose of 10 *μ*mol/kg (Figure 5(c)).

#### 3.6. Effects on IgG_2a_ and IgE in the Serum and BALF

**Figure 6:**
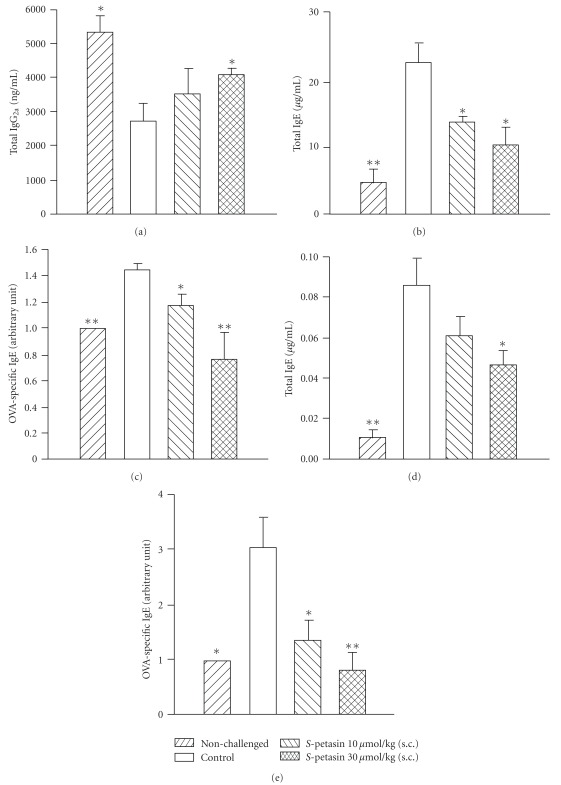
Effects of *S*-petasin (10–30 *μ*mol/kg, s.c.) on the total IgG_2a_ (a) level in the serum, and total IgE (b, d) and OVA-specific IgE (c, e) levels in the serum (b, c) and BALF (d, e) of sensitized mice which received aerosolized MCh (6.25–50 mg/mL) 2 days after secondary allergen challenge. *P < .05 and **P < .01, compared to the vehicle (control). Each value represents the mean ± SEM. The number of mice in each group was 10.

Compared to non-challenged mice, the total IgG_2a_ levels in the serum of control sensitized and challenged mice were significantly reduced. *S*-Petasin (30 *μ*mol/kg, s.c.) significantly reversed this reduction (Figure 6(a)). Levels of total and OVA-specific IgE in the serum and BALF of control sensitized and challenged mice were significantly enhanced compared to non-challenged mice (Figures 6(b)–6(e)). *S*-Petasin (10–30 *μ*mol/kg, s.c.) dose-dependently and significantly suppressed these enhancements with the exception of total IgE in the BALF at 10 *μ*mol/kg.

#### 3.7. No Effect on Xylazine/Ketamine-Induced Anesthesia

**Figure 7:**
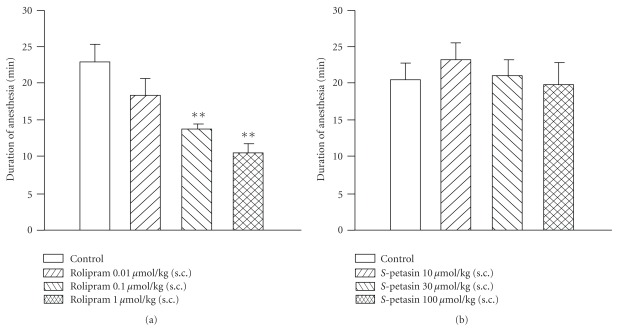
Effects of subcutaneously administered rolipram (a) and *S*-petasin (b) on the duration of xylazine (10 mg/kg, i.p.)/ketamine (70 mg/kg, i.p.)-induced anesthesia in mice. Rolipram or *S*-petasin was administered 15 min or 1 h before anesthesia, respectively. **P < .01, compared to the vehicle (control). Each value represents the mean ± SEM. The number of each group was 5–11.

The durations of xylazine/ketamine-induced anesthesia in control (vehicle) mice of *S*-petasin- and rolipram-treated group were 20.6 ± 2.2 (*n* = 10) and 22.8 ± 2.7 min (*n* = 11), respectively. Rolipram (0.1–1 *μ*mol/kg, s.c.) dose-dependently and significantly shortened the duration (Figure 7(a)). In contrast to rolipram, *S*-petasin (10–100 *μ*mol/kg, s.c.) did not affect the duration (Figure 7(b)).

#### 3.8. Inhibition of OVA-Induced Contractions In Vitro

**Figure 8:**
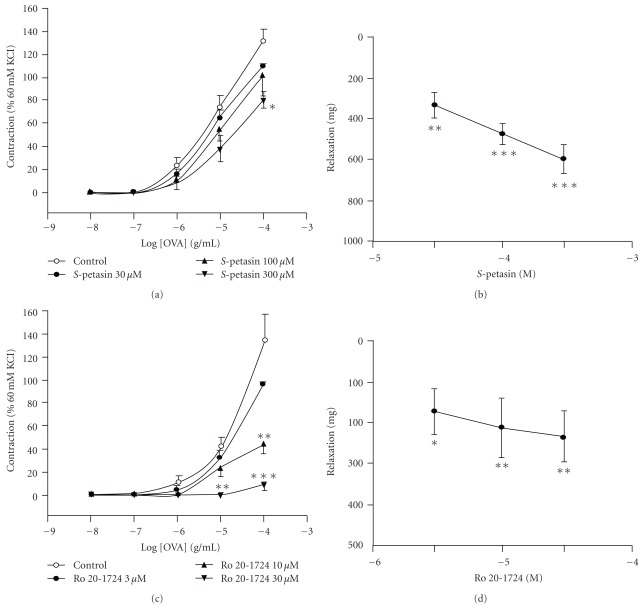
Effects of *S*-petasin (a, b) and Ro 20-1724 (c, d) on cumulative OVA-induced contractions (a, c) and baseline tension (b, d) in isolated sensitized guinea pig trachealis. *P < .05, **P < .01, and ***P < .001, compared to the control (vehicle). Each value represents the mean ± SEM (*n* = 5–9).

In isolated sensitized guinea pig trachea, 60 mM KCl evoked a contraction and increased tension to 997 ± 93 mg (*n* = 36), which was set as 100%. OVA (0.01–100 *μ*g/mL) alone concentration-dependently enhanced the tension from the baseline to 130.8 ± 11.6% (*n* = 9) of the 60 mM KCl-induced contractions (Figure 8(a)). The log concentration-response curve of OVA was unaltered by 1 *μ*M nifedipine, a selective voltage-dependent calcium channel blocker [37] (data not shown). *S*-Petasin (30–100 *μ*M) also did not significantly inhibit OVA (100 *μ*g/mL)-induced contractions compared to the control (vehicle), although *S*-petasin at 300 *μ*M did (Figure 8(a)). The vehicle of *S*-petasin did not affect the baseline tension or OVA (100 *μ*g/mL)-induced maximal contractions (data not shown). However, *S*-petasin (30–300 *μ*M) concentration-dependently and significantly relaxed the baseline tension (Figure 8(b)). In contrast, Ro 20-1724 (10–30 *μ*M) concentration-dependently inhibited OVA (10–100 *μ*g/mL)-induced contractions. Ro 20-1724 at 30 *μ*M even significantly inhibited OVA (10 *μ*g/mL)-induced contractions (Figure 8(c)). Similarly to *S*-petasin, Ro 20-1724 (3–30 *μ*M) concentration-dependently and significantly relaxed the baseline tension (Figure 8(d)).

## 4. Discussion

In the present results, *S*-petasin suppressed all types of inflammatory cells examined, including total inflammatory cells, lymphocytes, neutrophils and eosinophils, but not macrophages, in the BALF of sensitized and challenged mice. The reason that macrophages were unaffected by *S*-petasin is unclear. Allergic asthma is a chronic respiratory disease characterized by AHR, mucus hypersecretion, bronchial inflammation and elevated IgE levels. T-helper type 2 (Th2) cells, together with other inflammatory cells such as eosinophils, B cells and mast cells, have been proposed as playing critical roles in the initiation, development and chronicity of this disease [38]. One hypothesis emphasizes an imbalance in Th cell populations favoring expression of Th2 over Th1 cells. Cytokines released from Th2 cells are IL-4, -5, -6, -9 and -13, and those from Th1 cells are IL-2 and -12, IFN-*γ* and TNF-*α* [39, 40]. In the present results, *S*-petasin suppressed levels of IL-2, -4 and -5, IFN-*γ* and TNF-*α*, suggesting that *S*-petasin suppresses both Th1 and Th2 cells. This inhibitory effect of *S*-petasin on both Th1 and Th2 cells is similar to that of AWD 12-281, a selective PDE4 inhibitor [41]. Th1 and Th2 cells have, respectively, been implicated in autoimmune and atopic diseases [42]. Therefore AWD 12-281 is currently under clinical evaluation for the topical treatment of atopic dermatitis [41].

IL-4 and -13 have been shown to induce AHR in mouse asthma models [43, 44]. IL-4 has three primary effects. First, IL-4 promotes B cell differentiation to plasma cells that secrete antigen-specific IgE antibodies. Second, IL-4 promotes mast cell proliferation. Third, increased IL-4 upregulates endothelial cell expression of adhesion molecules for eosinophils [45]. IL-5 mobilizes and activates eosinophils, leading to the release of a major basic protein, cysteinyl-leukotrienes, and eosinophil peroxidase that contribute to tissue damage and AHR [44, 46]. Phosphoinositide 3-kinase *δ* (p110*δ*) was shown to play a crucial role in the development, differentiation and antigen receptor-induced proliferation of mature B cells [47, 48], and inhibition of p110*δ* attenuates allergic airway inflammation and AHR in a murine asthma model [48, 49]. In addition, IL-4 and -13 are important in directing B cell growth, differentiation and secretion of IgE [50]. However, IFN-*γ* released from Th1 cells preferentially directs B cell switching of IgM to IgG_2a_ and IgG_3_ in mice [51, 52]. The biological activities of IgE are mediated through the high-affinity IgE receptor (Fc*ε*RI) on mast cells and basophils. Cross-linking of the Fc*ε*RI initiates multiple signaling cascades leading to cellular degranulation and activation [53, 54]. Recently, p110*δ* activity was reported to be critical for allergen-IgE-induced mast cell degranulation and release of cytokines [55]. Inhibition of p110*δ* therefore attenuates the production of IgE as well as allergen-IgE-induced mast cell activation during allergic inflammation. The calcium channels in mast cell membranes were proposed to differ from those in cardiovascular tissues [56], which are sensitive to nifedipine. In the present *in vitro* results, *S*-petasin (30–100 *μ*M), but not at 300 *μ*M which is beyond a physiological condition, did not inhibit cumulative OVA-induced contractions of isolated sensitized guinea pig trachealis, which were unaffected by 1 *μ*M nifedipine, suggesting that *S*-petasin does not inhibit degranulation or activation of mast cells [57]. Indeed, *S*-petasin was reported to be an L-type voltage-dependent Ca^2+^ channel blocker in rat aortic smooth muscle cells [58, 59] and in mouse NG 108-15 neuronal cells [60]. In these neuronal cells, a higher concentration (100 *μ*M) of *S*-petasin was also reported to inhibit the delayed rectifier K^+^ current in a time-dependent manner, suggesting that such a blockage does not seem to be instantaneous, but develops with time after the channels are opened [60]. The blockage by *S*-petasin may contribute to the regulation of neuronal activity, but not to relaxation of the trachea-bronchial tree. Owing to the different sensitivities between vascular and tracheal smooth muscle cells, we demonstrated that in the presence of nifedipine (10 *μ*M), *S*-petasin or *S*-isopetasin (100 *μ*M each) further relaxed the CCh-induced precontractions in isolated guinea pig trachealis via cAMP-PDE inhibition or antimuscarinic effects, respectively [11]. The extents of relaxation by nifedipine and these two sesquiterpenes (*S*-petasin and *S*-isopetasin) were 20%–30%, and 70%–80%, respectively. In other words, there are two mechanisms, at least, in tracheal relaxation by *S*-petasin or *S*-isopetasin. In the present results, *S*-petasin and Ro 20-1724 concentration-dependently relaxed the baseline in isolated sensitized guinea pig trachealis, suggesting that *S*-petasin has a bronchodilatory effect. This aspect is strongly supported by our previous reports [9, 11]. In addition, *S*-petasin dose-dependently and significantly suppressed total and OVA-specific IgE levels in the serum and BALF, and enhanced the level of total IgG_2a_ in the serum of sensitized and challenged mice, suggesting that *S*-petasin appears to have immunoregulatory effects.

Selective PDE4 inhibitors specifically prevent the hydrolysis of cAMP, a 3′,5′-cyclic nucleotide, and therefore have broad anti-inflammatory effects such as inhibition of cell trafficking, and cytokine and chemokine release from inflammatory cells. The second-generation PDE4 inhibitors, cilomilast and roflumilast, have reached the clinical trial stage and have exhibited some beneficial effects in treating asthma and COPD [61]. The effectiveness of these PDE4 inhibitors may be limited by their clinical potency when using doses that have minimal adverse effects on headaches, diarrhea, nausea and abdominal pain. The PDE4_H_/PDE4_L_ ratios of cilomilast and roflumilast were reported to be 1 [62] and 3 [29], respectively, which are considerably greater than that (0.01–0.001) of rolipram [30]. Owing to its low PDE4_H_/PDE4_L_ ratio, cilomilast was discontinued for use against asthma after phase II clinical trials in 2003 [61]. In terms of tolerability over 6 months with 15 mg twice daily for COPD in a phase III study, cilomilast was reported to be associated with higher frequencies of diarrhea and nausea than a placebo [61]. Roflumilast is still being evaluated for asthma and COPD in phase III clinical trials at present, and is reported to reduce these adverse effects after longer-term treatment at 0.5 mg once daily [61]. The PDE4_H_/PDE4_L_ ratio of AWD 12-281, another selective PDE4 inhibitor, was calculated to be approximately 11 [63]. AWD 12-281 has been undergoing clinical development phase IIa trials for COPD, and has been reported to be a unique potential drug for the topical treatment of asthma and COPD [64]. Recently, AWD 12-281 was reported to be a very promising drug candidate for treating lung inflammation when administered by inhalation and for treating atopic dermatitis [65].

**Figure 9:**
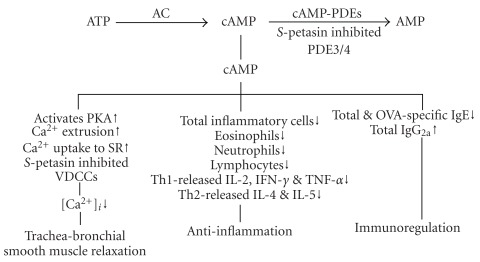
Mechanisms of action of *S*-petasin. *S*-Petasin mainly inhibits PDE3/4 activities and results in increase of cAMP, which activates cAMP-dependent protein kinase (PKA) and increases calcium extrusion from intracellular space and uptake to sarcoplasmic reticula (SR). *S*-Petasin was also reported to inhibit voltage-dependent calcium channels (VDCCs). Therefore, *S*-petasin largely decreases the concentration of intracellular calcium ([Ca^2+^]*_i_*) and results in trachea-bronchial smooth muscle relaxation. The increased cAMP also has anti-inflammatory and immunoregulatory effects. AC, adenylate cyclase. Up and down arrows indicate increases and decreases, respectively.

In the present results, the PDE4_H_ value of *S*-petasin was >300 *μ*M, suggesting that it has a low affinity for HARBSs of brain cell membranes. Thus the PDE4_H_/PDE4_L_ ratio of *S*-petasin was >17, which is greater than that of AWD 12-281. In addition, *S*-petasin did not affect xylazine/ketamine-induced anesthesia. This result is consistent with its low affinity for HARBSs of brain cell membranes. However, rolipram, a selective PDE4 inhibitor, reversed the anesthesia. The reversing effect may occur through presynaptic *α*_2_-adrenoceptor inhibition [66], because MK-192, an *α*_2_-adrenoceptor antagonist, was reported to reverse xylazine/ketamine-induced anesthesia in rats [67] and trigger vomiting in ferrets [66]. In contrast, clonidine, an *α*_2_-adrenoceptor agonist, prevented emesis induced by PDE4 inhibitors in ferrets [66]. In contrast to rolipram or AWD 12-281, *S*-petasin is a dual PDE3/4 inhibitor. Thus, the present results for *S*-petasin at least partially explain why *Petasites formosanus* is used as a folk medicine to treat asthma in Taiwan. However, whether *S*-petasin has other adverse effects or has good bioavailability after oral administration should be further evaluated. In summary, PDE3/4 inhibition and VDCC blockage are the main mechanisms of action of *S*-petasin (Figure 9).

## Figures and Tables

**Table 1:** The IC_50_ and *K*_i_ (*μ*M) values of *S*-petasin and reference drugs on PDE isozymes 1–5.

Test compound	PDE isozyme				
	1	2	3	4	5
*S*-Petasin					
IC_50_	>100 (*n* = 3)	>100 (*n* = 3)	25.5 ± 1.5 (*n* = 4)	17.5 ± 2.4 (*n* = 6)*	>100 (*n* = 3)
*K*_i_	ND	ND	25.3 ± 2.0 (*n* = 4)	18.1 ± 1.7 (*n* = 4)*	ND
Reference drugs^a^					
IC_50_	36.5 ± 8.8 (*n* = 4)	6.5 ± 1.0 (*n* = 4)	1.5 ± 0.2 (*n* = 8)	6.9 ± 0.6 (*n* = 4)	4.1 ± 1.1 (*n* = 4)
*K*_i_	ND	ND	1.6 ± 0.2 (*n* = 4)	4.2 ± 0.7 (*n* = 4)	ND

All values are expressed as the mean ± SEM (*n*), and *n* is the number of experiments. ND, not determined.

^a^Reference drugs for PDE isozymes 1, 2, 3, 4 and 5 were vinpocetin, EHNA, milrinone, Ro 20-1724 and zaprinast, respectively.

*P < .05, compared to the corresponding value of PDE3.
